# Clonal Expansion during *Staphylococcus aureus* Infection Dynamics Reveals the Effect of Antibiotic Intervention

**DOI:** 10.1371/journal.ppat.1003959

**Published:** 2014-02-27

**Authors:** Gareth McVicker, Tomasz K. Prajsnar, Alexander Williams, Nelly L. Wagner, Michael Boots, Stephen A. Renshaw, Simon J. Foster

**Affiliations:** 1 Krebs Institute, University of Sheffield, Western Bank, Sheffield, United Kingdom; 2 Department of Molecular Biology and Biotechnology, University of Sheffield, Western Bank, Sheffield, United Kingdom; 3 MRC Centre for Developmental and Biomedical Genetics, University of Sheffield, Western Bank, Sheffield, United Kingdom; 4 Department of Infection and Immunity, University of Sheffield, Western Bank, Sheffield, United Kingdom; 5 Biosciences, University of Exeter, Cornwall Campus, Penryn, United Kingdom; University of Tubingen, Germany

## Abstract

To slow the inexorable rise of antibiotic resistance we must understand how drugs impact on pathogenesis and influence the selection of resistant clones. *Staphylococcus aureus* is an important human pathogen with populations of antibiotic-resistant bacteria in hospitals and the community. Host phagocytes play a crucial role in controlling *S. aureus* infection, which can lead to a population “bottleneck” whereby clonal expansion of a small fraction of the initial inoculum founds a systemic infection. Such population dynamics may have important consequences on the effect of antibiotic intervention. Low doses of antibiotics have been shown to affect *in vitro* growth and the generation of resistant mutants over the long term, however whether this has any *in vivo* relevance is unknown. In this work, the population dynamics of *S. aureus* pathogenesis were studied *in vivo* using antibiotic-resistant strains constructed in an isogenic background, coupled with systemic models of infection in both the mouse and zebrafish embryo. Murine experiments revealed unexpected and complex bacterial population kinetics arising from clonal expansion during infection in particular organs. We subsequently elucidated the effect of antibiotic intervention within the host using mixed inocula of resistant and sensitive bacteria. Sub-curative tetracycline doses support the preferential expansion of resistant microorganisms, importantly unrelated to effects on growth rate or *de novo* resistance acquisition. This novel phenomenon is generic, occurring with methicillin-resistant *S. aureus* (MRSA) in the presence of β-lactams and with the unrelated human pathogen *Pseudomonas aeruginosa*. The selection of resistant clones at low antibiotic levels can result in a rapid increase in their prevalence under conditions that would previously not be thought to favor them. Our results have key implications for the design of effective treatment regimes to limit the spread of antimicrobial resistance, where inappropriate usage leading to resistance may reduce the efficacy of life-saving drugs.

## Author Summary

*Staphylococcus aureus* is a major cause of human disease, made even more notable due to the spread of antibiotic resistance. We used a combination of animal models to study the spread of bacteria between organs during an infection and the resulting effect of antibiotic intervention. We found that *S. aureus* infection is highly clonal, following a “bottleneck” in which very few bacterial cells found each abscess. Despite previous *in vitro* research, the effect of antibiotics on *S. aureus* infection was poorly understood. We utilized our systemic infection models to study intervention with sub-curative antibiotic doses, such as one might encounter upon failing to complete an antibiotic course. We have shown that such doses are able to support the preferential expansion of antibiotic-resistant organisms during a mixed infection. This selection is due to the clonal pattern of infection, occurring despite a lack of effect on growth rate or on the spontaneous generation of resistance. Furthermore, it is generic to multiple pathogen species, including *Pseudomonas aeruginosa*, and antibiotic classes, such as with methicillin-resistant *S. aureus* (MRSA) in the presence of oxacillin. Given the current debate in the field, our results have important implications for the design of properly-controlled treatment regimes.

## Introduction

*Staphylococcus aureus* is an opportunistic human pathogen that causes skin and tissue abscesses, occasionally leading to severe systemic illness and death [1]. Whilst the process of lesion formation itself is becoming better defined [2], the dynamics of a bacterial population within the host during infection are far less well understood. Whilst it is likely that infection progression and tissue tropism vary between different strains of *S. aureus* (such as those that cause osteomyelitis or keratitis [3], [4]), research on zebrafish embryos and mice has indicated an important role of host phagocytes in the infection process [5]–[8]. This leads to an infection “bottleneck” in which a small fraction of the initial inoculum goes on to found a systemic infection. Previous studies have identified bottleneck phenomena for a range of bacterial species [9], [10].

*S. aureus* is infamous for its rapid development of antibiotic resistance, which has grown increasingly more relevant with the widespread use of antimicrobials in agriculture and medicine. Alarmingly, where once it was restricted to health-care settings, drug-resistant *S. aureus* is now also found in the wider community [11], [12]. Bacteria resistant to antibiotics such as methicillin and tetracycline have been shown to colonize humans in contact with antibiotic-treated livestock [13], [14]. The emergence of antibiotic resistance in staphylococcal species has been the subject of much study; classically, studies and analyses have focused on the generation of resistance, persistence or tolerance due to advantageous mutations in a sensitive bacterial population challenged by high levels of antibiotics [15]–[17]. Development of resistance may come at a fitness cost [18], although *S. aureus* may be able to circumvent this cost via methods such as phenotypic switching [19]. Likewise, resistance to some drugs (including tetracycline and oxacillin) is known to be inducible [20], [21].

Of current interest is the effect of sub-curative concentrations of antibiotics; that is, treatment that confers no significant improvement in the wellbeing of the host. Such low levels of antibiotics might be generated during failure to complete a treatment regime, or encountered via the hospital environment or in agriculture. The effects of a reduced antibiotic dose are the focus of several recent studies and reviews, again largely concerning the generation of mutants [22]–[24]. This area of research spans agriculture [13], [14], [25], [26], medicine [27], [28] and food safety [29] and examines multiple bacterial genera. For example, *Pseudomonas aeruginosa* has been shown to acquire antibiotic resistance more rapidly in the presence of low levels of antibiotic [30], and is known to be associated with staphylococcal infection, particularly in the lungs of cystic fibrosis patients [31]. Selection of antibiotic-resistant clones during low-level treatment *in vivo*, however, has not been directly examined.

In this study we use a set of isogenic, antibiotic-resistant strains to investigate the bacterial population dynamics within both zebrafish embryo and murine systemic infection models. Using these strains, we elucidate the effect of low levels of antibiotics on bacteria *in vivo*, showing that it is possible to confer an advantage to an antibiotic-resistant strain, even at levels of antibiotics that do not affect the growth rate of an antibiotic-sensitive strain or its ability to cause a lethal infection.

## Results

### Construction and characterization of antibiotic-resistant *Staphylococcus aureus* strains

In order to enhance the ability of infection dynamics studies to examine disease progression in both zebrafish embryos and mice, a set of three antibiotic-resistant *S. aureus* strains were constructed in the SH1000 background: erythromycin/lincomycin-resistant, EryR (GMSA015); kanamycin-resistant, KanR (GMSA016); and tetracycline-resistant, TetR (GMSA017). Growth of the three strains *in vitro* under favorable conditions exactly matched the wild type parent SH1000 (Figure S1A). Furthermore, virulence assays in the zebrafish embryo model (1500 CFU administered into the circulation at 30 hours post fertilization) resulted in the pattern of mortality one would expect from wild type bacteria (Figure S1B). Resistance cassettes were then transduced into a second strain background, NewHG, creating strains GMSA021, GMSA022 and GMSA023 (EryR, KanR and TetR respectively). NewHG is a Newman derivative in which the gene encoding regulator SaeS has been repaired, resulting in a level of expression of *saeS* and downstream virulence factors that matches many other *S. aureus* strains, including the community-acquired methicillin-resistant *S. aureus* (MRSA) lineage USA300, as opposed to the much higher levels of expression seen in the presence of the Newman allele [32].

### Three-strain infection dynamics in the zebrafish embryo model

**Figure 1:**
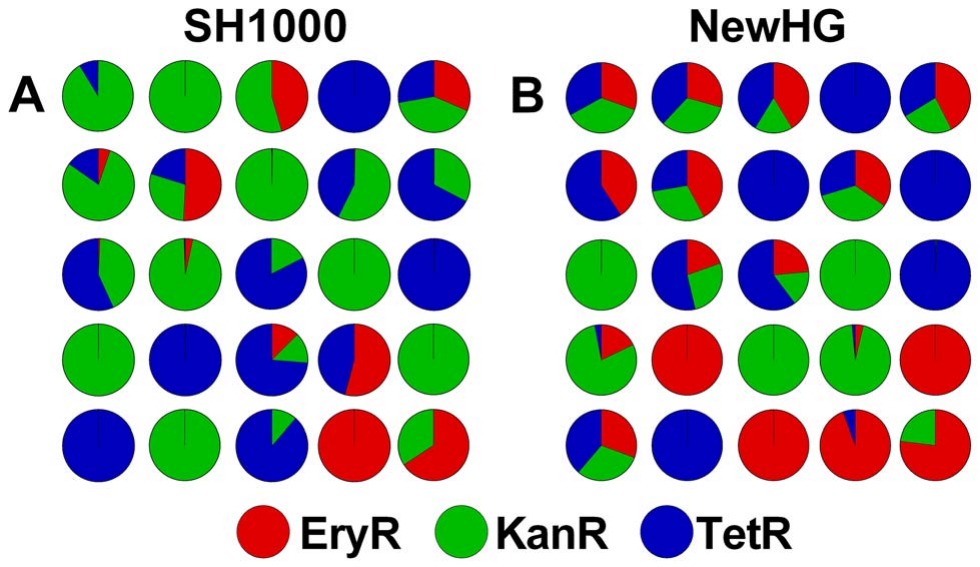
The stochastic distribution of bacterial strains *post mortem* in zebrafish embryos. Embryos were infected with a 1∶1∶1 mixture of three antibiotic resistance-marked, but otherwise isogenic, strains. Each pie chart represents a single embryo infected with *S. aureus* (A) SH1000 variants or (B) NewHG variants. Total bacterial load in each case was approximately 10^6^ CFU.

Previous work in the zebrafish embryo model suggested that growth of bacteria *in vivo*, leading to lethality, occurs after a biological “bottleneck” that often results in clonal expansion of a single strain [5]. This phenomenon was demonstrated by infecting zebrafish embryos 30 hours post fertilization with 1500 CFU of a 1∶1∶1 mixture of the three SH1000 strains (GMSA015-017) and enumerating the strains present in each embryo *post mortem* (Figure 1A). Infection with NewHG strains (GMSA021-023) gave similar results (Figure 1B). In both backgrounds, differential clonal expansion produced cases wherein either one or two strains out of three predominated (alongside cases wherein all three strains grew equally). There was no preference for the growth of any particular strain over its competitors, with all three antibiotic resistance cassettes proving equally capable of being selected (*p*>0.1), in agreement with previously published results [5]. Furthermore, we isolated clones that had been selected *in vivo* and, after mixing with “naïve” bacteria, reintroduced them into the zebrafish model. We found that there was no increase in the selection of these clones relative to their naïve counterparts over three successive passages, suggesting a lack of adaptive mutations, as expected [5]. Therefore *in vivo* clonal population expansion of the three strains is likely to be entirely stochastic and, hence, endpoint enumeration of these strains is a suitable and reliable method to investigate *in vivo* dynamics.

### A mixed, three-strain infection reveals complex pathogen dynamics within the murine host

It is known that intravenous *S. aureus* infection results in kidney abscesses [2], [33] and that these likely originate from a small number of founding bacteria [5]. Modeling of infection dynamics has been performed in the murine model using other organisms, including *Salmonella enterica* [9], *Bacillus anthracis* [10] and *Yersinia pseudotuberculosis* [34]. Herein, a three-strain inoculum was used to investigate the dynamics of systemic staphylococcal disease. Female BALB/c mice were infected intravenously at 7–8 weeks of age with a mixed inoculum of NewHG strains (GMSA021-023) at a 1∶1∶1 ratio totaling 1×10^7^ CFU. At 30 minutes and then at 2, 6, 12, 24, 48 and 72 hours post infection, mice were sacrificed and their visceral organs (kidneys, liver, spleen, heart and lungs) were harvested for bacterial enumeration by homogenization, serial dilution and plating on selective media (n = 8–10 mice per time-point). Bacterial numbers in the blood were found to be consistently low, approaching the limit of detection at all time-points, and are therefore not included in the analysis. Pilot data indicated that the bacteria were unlikely to be found at other sites (including brain, thigh muscle/bones and the site of infection, i.e. tail).

**Figure 2:**
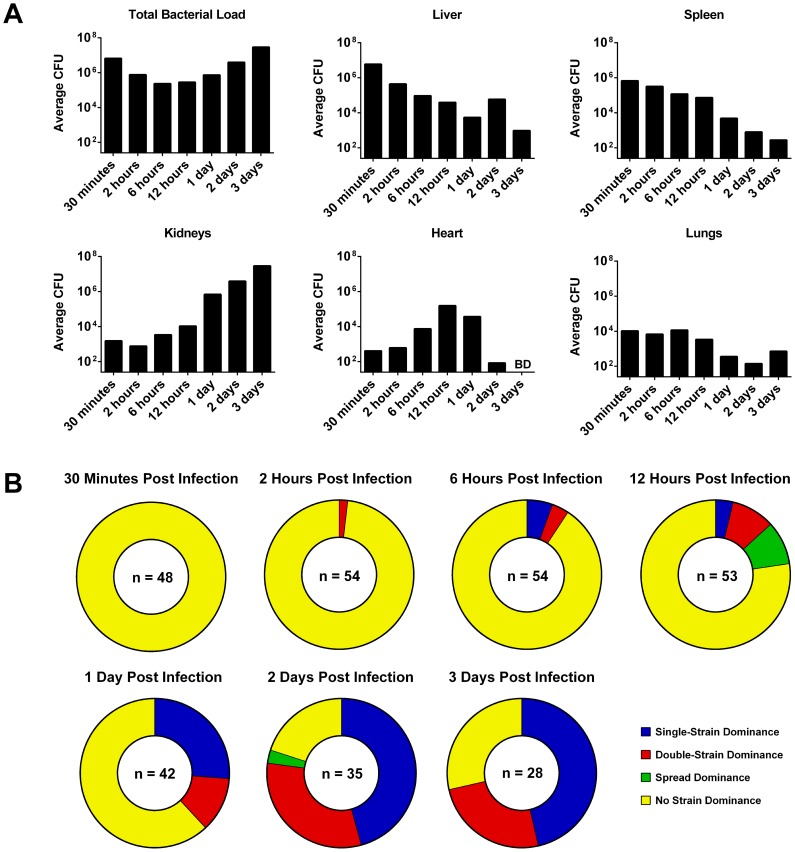
The bacterial strain distribution in mouse organs at various time-points post infection. Mice were infected with a 1∶1∶1 mixture of three marked *S. aureus* NewHG strains (n = 8–10 mice per time-point). (A) The change in total CFU load over time in each organ (BD: below limit of detection). (B) The number of organs at each time-point that show either single-strain dominance (one strain ≥100× the other two), double-strain dominance (two strains ≥100× the third), spread dominance (highest and lowest strains differ by ≥100×, with the middle strain within 100× of both) or no dominance (all strains are within 100× of one another).

A large fraction of the bacterial inoculum was restricted to the liver and spleen for several hours post infection. Neither extreme clonality nor an overall increase in bacterial numbers were observed at this stage, indicating that little bacterial growth was occurring, or that there was an equilibrium between bacterial growth and killing by the host (Figure 2). As the infection progressed, bacteria were found transiently in the heart (peaking at 12 hours post infection) and then in increasingly large numbers in the kidneys (peaking at 2–3 days post infection) (Figure 2A). Bacteria in the kidneys showed a high degree of clonality, in most cases representing only one or two strains out of three. Occasionally, highly-clonal bacterial populations were found in the liver at later time-points. This dramatic increase in clonality over time is shown in Figure 2B. Data for individual organs at each time-point are given in supplementary Figure S2. One mouse (labelled “*” in Figure S2) was sacrificed for humane reasons due to ill health at 42 hours post infection and was excluded from the general analysis.

### Sub-curative concentrations of tetracycline alter the dynamics of a staphylococcal infection in zebrafish

Clonal expansion of bacteria has important implications during mixed-strain infections, especially when a drug-resistant mutant is present among a population of sensitive organisms, and the zebrafish infection model allowed us to investigate this process. The antibiotic dose required to produce a significant reduction in mortality in the zebrafish embryo model can, in some cases, approach fifty times the *in vitro* MIC. It was hypothesized that a low antibiotic dose, i.e. one that produces no response in fish infected with a drug-sensitive strain, might still offer a preferential advantage to drug-resistant bacteria, as has been observed *in vitro* [23].

In order to define an appropriate drug concentration, tetracycline response experiments were conducted, whereupon embryos infected with 1500 CFU SH1000 EryR (GMSA015) were immersed in sterile E3 medium containing a range of antibiotic doses for the duration of the experiment (Figure S3A).While 50 µg/ml tetracycline produced a highly significant, curative response (*p* = 0.0003), 10 µg/ml tetracycline showed a trend towards curing (*p* = 0.0753) and 5 µg/ml was entirely sub-curative (*p* = 0.2181). We therefore chose 2.5 µg/ml tetracycline for subsequent experiments. Embryos treated with sub-curative 2.5 µg/ml tetracycline contained approximately 10^6^ CFU of EryR bacteria upon death (Figure S3B), demonstrating no reduction in terminal bacterial load at this dose. This control indicates that any observed skewing of the population in favor of a resistant strain at this antibiotic concentration would not result from failure of the sensitive strain to grow to normal levels *in vivo*. Lastly, bacterial growth kinetics were assayed *in vivo* using either the EryR or TetR strain injected alone into zebrafish embryos and either left untreated or treated with 2.5 µg/ml tetracycline. Neither strain showed significantly different growth kinetics *in vivo* in the presence of tetracycline (*p*>0.9) (Figure S4), indicating that the skewing effect is not simply due to a change in growth rate within the host organism. Growth rate controls were not performed *in vitro* because the antibiotic concentrations involved would not correspond well with *in vivo* work. Experiments performed by others show that low antibiotic doses can indeed select for resistant mutants *in vitro* over the long term [23], but that the effect requires a far greater number of generations than exhibited by bacterial growth in our *in vivo* experiments.

**Figure 3:**
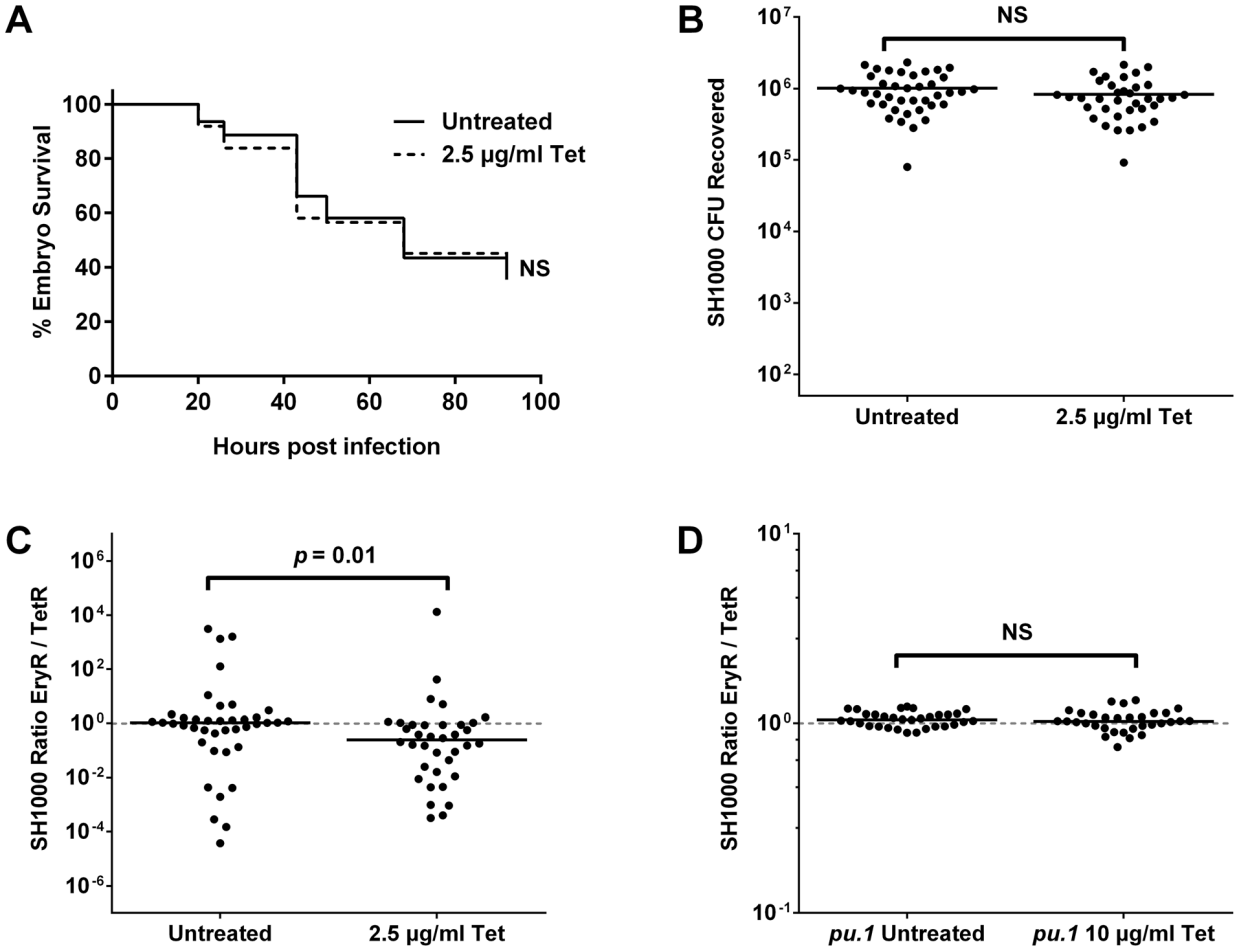
The effect of a sub-curative antibiotic dose on zebrafish embryos infected with *S. aureus* SH1000. Embryos were infected with a 1∶1 mixture of SH1000 EryR∶TetR bacteria and treated with 2.5 µg/ml or 10 µg/ml tetracycline as indicated. (A) Zebrafish mortality curve (n = 60–65 per group). (B) Total terminal bacterial load per embryo. (C) Terminal EryR/TetR strain ratio per embryo. (D) Terminal EryR/TetR strain ratio per *pu*.1 morphant (phagocyte-depleted embryo). Solid lines indicate mean (B) and median (C, D) values.

To ascertain whether a sub-curative dose of antibiotic was indeed able to influence bacterial population dynamics, embryos were infected with a total of 1500 CFU SH1000 EryR and TetR (GMSA015 and GMSA017 mixed 1∶1), and treated with sub-curative 2.5 µg/ml tetracycline. Treated and untreated embryos showed no difference in mortality (Figure 3A, *p* = 0.7412) or in total bacterial numbers per embryo upon death (Figure 3B, *p* = 0.1452). There were, however, significant differences in the ratios between the strains isolated from treated and untreated embryos (Figure 3C, *p* = 0.0143). Lowering the tetracycline dose to 1 µg/ml abolished this effect (Figure S3C, *p* = 0.1008), implying a narrow window (approximately four-fold) in which the antibiotic dose is low enough to be statistically sub-curing but high enough to influence population dynamics. Bacteria recovered from these experiments were invariably found to be resistant to a single antibiotic alone, therefore the change in output ratio is not due to the spontaneous generation of resistance among the sensitive population (as bacteria that had acquired resistance to tetracycline in this way would remain erythromycin-resistant). Phagocyte depletion of infected zebrafish, which has been previously shown to prevent stochastic population variation [5], resulted in untreated and treated groups that were statistically similar even after treatment with a higher dose of 10 µg/ml tetracycline (Figure 3D; *p* = 0.4855), indicating that phagocytes play a role in the antibiotic skewing phenomenon, and again supporting the hypothesis that phagocytes are an important host niche during *S. aureus* infection.

### *In vivo* selection of resistant clones occurs among other strains, genera and antibiotic classes

**Figure 4:**
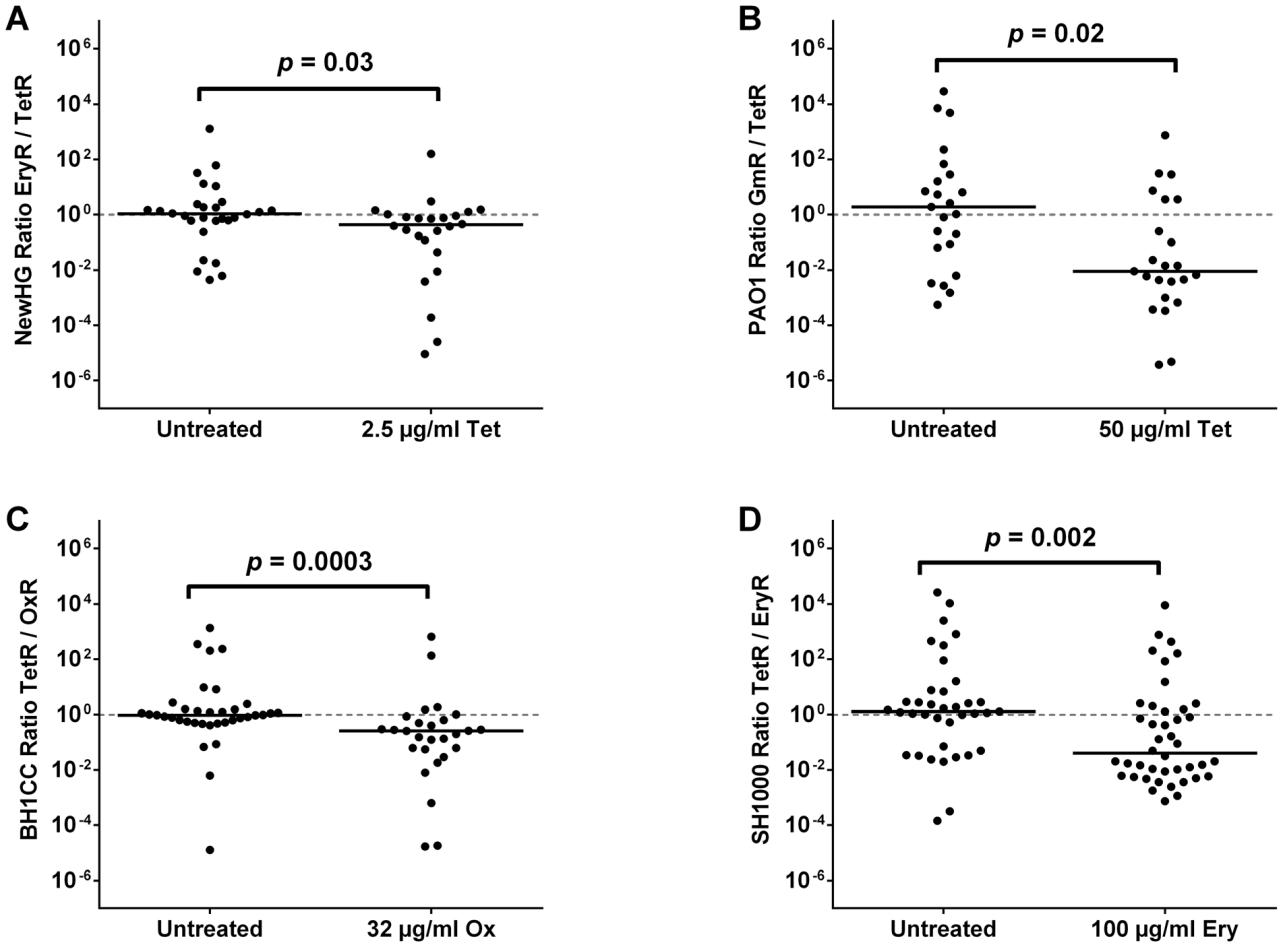
The effect of sub-curative antibiotic doses on zebrafish embryos infected with a variety of pathogens. Embryos were infected with a 1∶1 mixture of (A) *S. aureus* NewHG EryR∶TetR bacteria (treated with 2.5 µg/ml tetracycline), (B) *P. aeruginosa* PAO1-L GmR∶TetR bacteria (treated with 50 µg/ml tetracycline), (C) *S. aureus* BH1CC OxS∶OxR bacteria (treated with 32 µg/ml oxacillin) or (D) *S. aureus* SH1000 EryR∶TetR bacteria (treated with 100 µg/ml erythromycin). Terminal sensitive/resistant strain ratio per embryo is shown in each case. Solid lines indicate median values.

To discover whether the phenomenon is consistent among other *S. aureus* strain backgrounds, the same experiments were conducted using NewHG EryR and TetR (GMSA021 and GMSA023). 2.5 µg/ml tetracycline was again shown in pilot experiments to be non-curative in fish infected with the sensitive strain alone (Figure S5A, *p* = 0.5443). Results of the NewHG mixed population experiment (1500 CFU, 1∶1) were consistent with those obtained for SH1000, showing similar embryo mortality between the treated and untreated groups (Figure S5B, *p* = 0.4575), similar total CFU recovered from both groups (Figure S5C, *p* = 0.4068) and a statistically significant difference between the strain ratios (Figure 4A, *p* = 0.0296). As expected, this was abolished in phagocyte-depleted embryos at 10 µg/ml tetracycline (Figure S5D, *p* = 0.2956). Therefore the preferential selection of antibiotic-resistant bacteria at low levels of antibiotic is not limited to a single *S. aureus* strain.

The Gram-negative pathogen *Pseudomonas aeruginosa* is commonly isolated alongside *S. aureus* in the lungs of patients with cystic fibrosis [31] and is able to modulate *S. aureus* growth and virulence factor expression during polymicrobial wound infection [35]. To explore the possibility that extreme skewing of strain ratios extends to genera of bacteria other than *Staphylococcus*, two drug-resistant *P. aeruginosa* PAO1-L derivatives, gentamicin-resistant GmR (GMPA001) and tetracycline-resistant TetR (GMPA002), were constructed. As expected from previous work by others [36], [37], live PAO1 (but not heat-killed PAO1) is lethal to zebrafish embryos when injected into the circulation, even at infectious doses as low as 100 CFU (Figure S6A). *P. aeruginosa* has a naturally high intrinsic resistance to tetracycline and was therefore unaffected by all attempts to cure the infection (Figure S6B) using drug doses that were not harmful to the embryo (≤100 µg/ml). In mixed population experiments with 50 µg/ml tetracycline (200 CFU, 1∶1), it was again observed that a lack of significant effect on embryo mortality (Figure S6C, *p* = 0.9449) or terminal CFU load (Figure S6D, *p* = 0.4474) did not prevent the selection phenomenon from occurring (Figure 4B, *p* = 0.0169). It is noteworthy, however, that *P. aeruginosa* does not reach a specific bacterial load before embryo mortality (unlike *S. aureus*, which consistently reaches approximately 10^6^ CFU). Furthermore, in contrast to the *S. aureus* strains tested above, phagocyte depletion of the host does not abolish the selection phenomenon in *P. aeruginosa* (Figure S6E, *p*<0.0001). Thus, despite the difference between *S. aureus* and *P. aeruginosa* population kinetics (and therefore infection dynamics) *in vivo*, the two species exhibit the same phenomenon with regards to selection at sub-curative antibiotic doses.

It was hypothesized that the effect of sub-curative antibiotic doses on strain ratios might not be restricted to tetracycline. To test this, clinical MRSA isolate BH1CC and its methicillin-sensitive, tetracycline-resistant isogenic partner [38] were used. Experiments were carried out in an identical manner to tetracycline (i.e. immersion of embryos in antibiotic). It was found that 32 µg/ml oxacillin had no effect on the mortality of zebrafish infected with 1500 CFU of either the sensitive strain alone (Figure S7A, *p* = 0.5390) or a 1∶1 mixture of sensitive and resistant strains (Figure S7B, *p* = 0.2087). Although the total bacterial load upon death was marginally but significantly decreased using this dose of oxacillin (Figure S7C, *p*<0.0001), this may be explained by the bactericidal action of oxacillin versus the bacteriostatic nature of tetracycline used in previous experiments. Nonetheless, such a decrease had no effect on the disease state or mortality of the infected host. This sub-curative dose of oxacillin produced a highly significant skew towards the resistant strain (Figure 4C, *p* = 0.0003), indicating that the phenomenon is not restricted to tetracycline and is likely to be an important factor in the clinical treatment of mixed MRSA-MSSA infections. Curiously, the skewing effect was also evident to a minor but significant degree in phagocyte-depleted embryos (Figure S7D, *p* = 0.0018) treated with the same oxacillin dose, suggesting both phagocyte dependent and independent effects. Selection was not significant at 16 µg/ml oxacillin, suggesting a 2-fold concentration range for the effect (Figure S7E, *p* = 0.0964).

Interestingly, antibiotics have been proposed to share a common killing mechanism involving accumulation of reactive oxygen species (ROS) within bacterial cells, and subsequent death from oxidative stress, irrespective of the antibiotic target or direct mechanism of action [39]. However, recent reports have questioned the role of ROS in the activity of bactericidal antibiotics [40], [41]. Therefore, the important components of staphylococcal oxidative stress resistance AhpC and KatA [42] were tested for their role in resistant clone selection *in vivo* (Figure S7F). Since the virulence of the *katA ahpC* double mutant (strain KC043) was not affected by oxacillin relative to its SH1000 parent at all concentrations tested (*p*>0.74), it is unlikely that ROS play a role in antibiotic skewing in the *in vivo* infection model.

Erythromycin is another medically relevant drug which our strain constructs enabled us to test. In the zebrafish embryo model, however, erythromycin proved unable to affect the outcome of infection by immersion alone. Instead, the antibiotic dose was introduced by microinjection not more than two hours after bacterial infection (untreated controls were instead injected with PBS). Results showed no significant difference between treated and untreated groups when comparing mortality during a single-strain infection (Figure S8A, EryR *p* = 0.0804, TetR *p* = 0.9964) or during a mixed infection (Figure S8B, *p* = 0.2695), nor was there a significant difference in terminal bacterial load (Figure S8C, *p* = 0.4438). Yet, again, there was a significant difference in terminal strain ratios (Figure 4D, *p* = 0.0022). Similarly to tetracycline, no cross-resistance was observed in the strains recovered from embryos *post mortem*. Therefore, preferential selection of resistant strains (not associated with the spontaneous generation of resistant mutants) results from treatment with sub-curative doses of multiple antibiotic classes.

### *In vivo* selection of resistant clones occurs in the mammalian host

In order to extend our findings to mammalian infection, antibiotic skewing experiments were performed using tetracycline in the murine model. Briefly, mixed-strain infections were carried out as for the population dynamics experiments above, and mice were treated with low doses of tetracycline in their drinking water. Mice were sacrificed two days post infection, when the majority of bacteria were expected to be found in clonal abscesses within organs (i.e. kidneys, liver and spleen), which were assayed for bacterial load.

**Figure 5:**
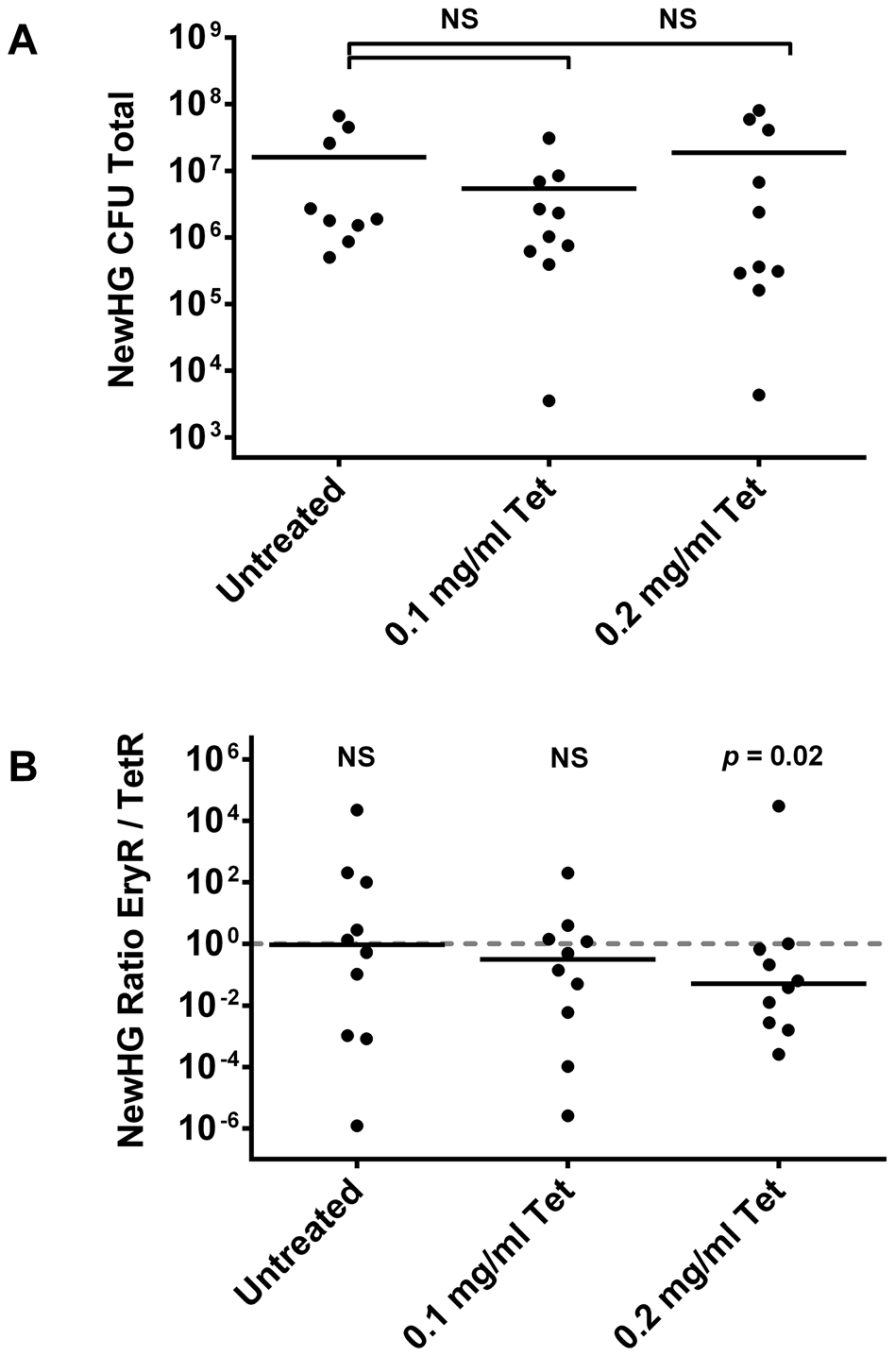
The effect of sub-curative antibiotic doses on mice infected with *S. aureus* NewHG. Mice were infected intravenously with a 1∶1 mixture of NewHG EryR∶TetR bacteria and were treated by replacing their drinking water with the indicated concentrations of tetracycline prior to sacrifice two days later. (A) Total CFU per mouse at experimental endpoint. (B) Total EryR/TetR strain ratio per mouse at experimental endpoint. Solid lines indicate mean (A) and median (B) values.

In preliminary experiments, the sub-curative tetracycline dose varied between animal groups. Two doses (0.1 mg/ml and 0.2 mg/ml) were therefore used in the final experiment alongside an untreated control (Figure 5). There was no significant difference in total CFU per mouse for either dose compared to the control (*p* = 0.1965 and *p* = 0.4194 respectively). Pooled data (i.e. the sum total of bacteria of each strain per mouse) showed a trend towards the resistant subpopulation. The variance in the untreated group compared to treated groups makes comparison by Mann-Whitney test highly misleading in this model without using hundreds of animals per group (an amount that would be impossible for both practical and ethical reasons); therefore, a binomial distribution was used to analyze these data. The underlying assumption of this conservative test is that in an untreated mouse, there is a 50% chance of skewing towards either strain (which is indeed what we observed; 5/10 towards TetR; *p* = 0.246). The comparison showed that whilst 0.1 mg/ml did not give a significantly non-stochastic result (6/10 TetR, *p* = 0.205), 0.2 mg/ml did (8/9 TetR and one mouse not skewed, *p* = 0.0176). Therefore a sub-curative antibiotic dose is able to cause preferential selection of resistant microorganisms during mammalian infection. Furthermore, as expected from our murine infection kinetics experiment, selection at this low antibiotic concentration was a result of growth in the kidneys (*p* = 0.0349) rather than liver (*p* = 0.2188) or spleen (*p* = 0.2344) (Figure S9).

## Discussion

Although *Staphylococcus aureus* is an important and increasingly antibiotic-resistant human pathogen, little is known about its disease progression or how antibiotic levels affect population dynamics. This is particularly pertinent for the understanding of *S. aureus* behavior in a hospital environment, in agriculture or even in the community at large, given the failure of some patients to complete prescribed antibiotic courses.

Construction of a set of isogenic strains that produce clonal lesions in the host demonstrated stochastic strain variance during zebrafish embryo infection. In the mouse it was shown that kidney abscesses form in the later stages of infection and each likely originates from a single bacterial founder. Interestingly, most of the initial inoculum is localized to the liver and spleen during the early stages of infection (up to approximately one day post intravenous administration). It is unknown whether bacterial cells founding renal abscesses were physically associated with the kidneys during this critical period, or whether individual bacteria “seed” the kidneys after travelling from another organ such as the liver, spleen, lungs, heart or blood at a later stage. Observation of a mouse that showed a dramatic, systemic infection of just two strains strongly implies that spread to secondary organs can occur after an initial period of clonal expansion. The variable speed at which growth occurs in the kidneys may be indicative of lesion founders “seeding” the kidneys at different times, especially in the case where two or more strains reach substantial but unequal numbers, given that no strain has a competitive advantage in terms of growth rate. In a peritoneal infection model, Rauch and colleagues reported parenchymal kidney abscesses that were identical to those observed post-intravenous infection, and that peritoneum-associated kidney surface abscesses were not associated with parenchymal abscesses [43]. Despite their role in controlling infection [44], host phagocytes may form the reservoir or delivery vehicle for *S. aureus* infection [5]–[8].

Although *S. aureus* colonization is not directly correlated with mortality [45], the presence of a mixed population of resistant and sensitive bacteria in the host provides a convenient “head start” for resistant bacteria to outgrow the sensitive strain and reach transmissible levels, should the patient become exposed to antibiotics. In the absence of treatment, however, resistance to antimicrobials has typically been thought to impose a fitness cost that enables the parent strain to outcompete antibiotic-resistant strains [18]. We have shown that this is not always the case, as even in the absence of apparent selective pressure, clonal expansion ensures that a pool of resistant bacteria can grow to dominant levels.

The use of antibiotics is the primary driving force behind the development of resistance [24]. It has been shown in *S. aureus* that vancomycin treatment is able to greatly increase a pool of resistant organisms inside a host [46] and that *de novo* resistance to tetracycline can arise when the drug is given orally at high levels [47]. Here, a combination of zebrafish embryo and murine systemic infection models were used to examine the role of sub-curative concentrations of antibiotics against multiple strains of *S. aureus* and a Gram-negative pathogen, *P. aeruginosa*, *in vivo*. A level of drug so low that it does not alter host mortality or bacterial load (i.e. a dose that is sub-curative) is able to produce a statistically significant skew in strain ratios towards a pre-existing resistant subpopulation. In *Escherichia coli*, low levels of antibiotics such as β-lactams promote stepwise mutations leading to resistance [22] and in *Pseudomonas aeruginosa*, sub-lethal concentrations of several antibiotics increase mutational frequency [30]. Sub-MIC doses of antibiotics have been demonstrated not only to contribute to the generation of resistant mutants, but also to allow a resistant organism to outcompete its sensitive competitors in long-term *in vitro* experiments [23].

Critically, our result is not dependent upon development of resistance in the sensitive subpopulation. Furthermore, selection of resistant clones is not simply due to retardation of growth of the sensitive bacteria caused by the antibiotic, as phagocyte-depleted zebrafish embryos (wherein bacteria grow exponentially from the outset of infection [48]) do not show a significant skew in favor of the resistant strain, nor did *in vivo* growth kinetics experiments reveal a significant difference. Since phagocyte-depleted embryos succumb to even a small bacterial dose within 18 hours [48], the restricted growth of the pathogen within the first 24 hours during our experiments suggests that all bacterial cells are captured by phagocytes immediately upon infection, therefore preferential phagocytosis is unlikely to be a factor. Instead, it is likely that the antibiotic confers an advantage to the resistant strain over the sensitive strain that allows it to better colonize the pre-expansion “niche” or somehow exploit that “niche” more effectively. This phenomenon may be limited to organisms whose mode of pathogenicity relies upon the stochastic selection of clones during infection, but is not restricted to a single species or class of antibiotic. It may be that such organisms are sensitive to low-dose treatment at a particular stage of their infectious cycle, in a niche-dependent fashion. Since the role of reactive oxygen species (ROS) in the action of antibiotics is currently under debate [39]–[41] and could provide an explanation for the skewing phenomenon, we examined the effect of ROS during treatment using an *S. aureus* strain (*ahpC katA*) defective in oxidative stress resistance [42]. Despite extreme sensitivity to oxidative stress the *ahpC katA* strain was no more susceptible to tetracycline *in vivo* than its isogenic parent, suggesting that ROS may not play a major role in a generalized antibiotic effect. It is well known that the bacterial transcriptome/proteome can fluctuate when exposed to sub-curative antibiotic doses [49]–[52], and this is an area of study that can be pursued in future work.

Environmental concentrations of antibiotics are commonly found in the ng/L to µg/L range except in extreme cases [53], [54], several orders of magnitude lower than the concentrations tested in our *in vivo* work. We have focused, therefore, on medical treatment of infection. The global spread of antibiotic resistance is a serious threat to human health that must be acted on, as it is inevitable that high-dose antimicrobial chemotherapy increases the selective pressure on the target organism. This has led to an argument that aggressive treatment regimes, such as those currently prescribed, should be reconsidered and that alternative patterns of treatment might be indicated [55]. As discussed above, however, it is becoming accepted that lower drug doses promote stepwise development of resistance and that aggressive drug use dramatically reduces the potential pool of clones from which resistance can develop. Given this information and our data showing that sub-curative antibiotic doses are still able to select for pre-existing resistant organisms, we suggest that removal of the sensitive bacteria before they can develop resistance is still the best strategy for control of microbial disease. Although we have not determined the concentrations of antibiotics active in host tissues, we note that the elimination half-lives of the antibiotics tested in this study vary considerably, from 30 minutes (oxacillin) to 6–12 hours (tetracycline) [56]. Coupled with our evidence that the sub-curative but selective dose range is two- to four-fold, we hypothesize that bacteria are likely to be in contact with a relevant drug concentration for ample time for selection to occur during treatment. Furthermore, many antibiotics including oxacillin and tetracycline share a renal route of elimination [56], which concurs with our murine experiments suggesting that the kidneys are important for the antibiotic effects discussed. The exposure time would be greatly amplified by “pulsed” antibiotic therapy. The effect of sub-curative antibiotics may seem modest, however, we note that the evolution of any organism does indeed occur as a result of modest competitive advantages. We believe that our results are indicative of a growing trend in the response of bacterial pathogens to antibiotics, and conclude that carefully prescribed, high-dose antimicrobial chemotherapy remains preferable over the alternatives at this time.

## Materials and Methods

### Ethics statement

Animal work (both mice and zebrafish) was carried out according to guidelines and legislation set out in UK law in the Animals (Scientific Procedures) Act 1986, under Project Licenses PPL 40/3123, PPL 40/3699 and PPL 40/3574. Ethical approval was granted by the University of Sheffield Local Ethical Review Panel.

### Bacterial strains and growth conditions

*Staphylococcus aureus* strains (Table S1) were grown using brain heart infusion (BHI) liquid or solid medium (Oxoid) at 37°C, supplemented with the following antibiotics where appropriate: kanamycin 50 µg/ml, tetracycline 5 µg/ml or erythromycin 5 µg/ml plus lincomycin 25 µg/ml (Sigma-Aldrich). *Pseudomonas aeruginosa* strains (Table S1) were grown using Luria-Bertani (LB) liquid or solid medium (Oxoid) at 37°C, supplemented with the following antibiotics where appropriate: tetracycline 125 µg/ml or gentamicin 20 µg/ml (Sigma-Aldrich).

### Construction of antibiotic-resistant *S. aureus* strains

The suicide vector pMUTIN4 [57] was used to integrate various antibiotic resistance cassettes downstream of the *lysA* gene (which encodes the terminal enzyme in the lysine biosynthetic pathway) in *S. aureus*. This provided a convenient method of screening to ensure that clones retained a wild type phenotype; undesired disruptions of *lysA* would result in lysine auxotrophy. Growth of clones in chemically defined minimal medium lacking lysine showed that the wild type *lysA* gene remained intact. After construction by standard PCR and restriction/ligation methods, plasmids were introduced into *S. aureus* RN4220 by electroporation, whereupon they integrated into the chromosome via homologous recombination. The resulting resistance markers were then transferred into other *S. aureus* strains as required by Φ11 transduction. pMUTIN4 provided erythromycin/lincomycin resistance (EryR), whereas pAISH1 [58] was used to integrate tetracycline resistance (TetR) and pGM072 (pMUTIN4 in which erythromycin resistance was replaced by the resistance cassette from pGL433 [59]) was used to integrate kanamycin resistance (KanR). BH1CC and its derivative [38] were kindly provided by James O'Gara (University College Dublin).

### Construction of antibiotic-resistant *P. aeruginosa* strains

*P. aeruginosa* PAO1-L derivatives were constructed by integration of mini-Tn7 to a neutral locus according to published protocols [60]. Plasmids containing mini-Tn7 marked with either a gentamicin or tetracycline resistance cassette (GmR or TetR respectively) were kindly provided, along with invaluable assistance, from Stephan Heeb (University of Nottingham).

### Zebrafish maintenance and microinjection

London wild-type (LWT) zebrafish embryos (bred in the MRC CDBG aquarium facilities at the University of Sheffield; see Ethics Statement) were used for all experiments and were incubated in E3 medium at 28°C according to standard protocols [61]. In order to obtain phagocyte-depleted embryos, morpholino-modified antisense oligomers against *pu.1* [62] were injected using a method described previously [48]. Anaesthetized embryos at 30 hours post fertilization were embedded in 3% w/v methylcellulose and injected individually using microcapillary pipettes filled with bacterial suspension of known concentration into the circulation, as previously described [48]. Following infection, embryos were kept individually in 100 µl E3 medium (with or without experimental antibiotics), observed frequently up to 92 hours post infection, dead embryos removed and numbers recorded at each time point.

### Intravenous mouse injections

Female BALB/c mice were purchased from Harlan (UK) and maintained at the University of Sheffield using standard husbandry procedures. The 7–8 week old mice were inoculated in the tail vein with 100 µl of *S. aureus* suspension in endotoxin-free PBS (Sigma) corresponding to 1×10^7^ CFU per mouse. Viable bacteria in the inoculum were plated on BHI (plus appropriate antibiotics) after serial decimal dilution to confirm the accuracy of the bacterial dose. Mice were monitored and sacrificed at various time-points according to experimental design.

### Determination of *in vivo* bacterial load

In order to recover bacteria from host tissues, whole zebrafish embryos or mouse organs were individually homogenized in a suitable volume of PBS using the PreCellys 24-Dual (Peqlab). Homogenates were serially diluted in PBS and plated on BHI (*S. aureus*) or LB (*P. aeruginosa*) agar supplemented with appropriate antibiotics to determine bacterial numbers.

### Statistical analysis

Survival experiments were evaluated using the Kaplan-Meier method. Comparisons between curves were performed using the log rank test. For comparisons between two CFU groups, a two-tailed, unpaired Student's *t*-test was used. For comparisons of strain ratios between two groups (e.g. treated and non-treated) in zebrafish embryos, a (non-parametric) Mann-Whitney *U* test was used. Analysis was performed using Prism version 6.0 (GraphPad) and statistical significance was assumed at p<0.05. In addition, analyses via generalized linear models, linear models on transformed data and Kruskall Wallace tests were performed and provided the same insights. Strain ratios in mice were analyzed by binomial distribution, as explained in Results. Figures show significance to 1 s.f. (*p*<0.05) or NS (*p*≥0.05), and indicate either mean (CFU comparison) or median (ratio comparison) values as appropriate.

## Supporting Information

Figure S1 **Comparison of fitness of antibiotic resistance-tagged SH1000 strains.** (A) Growth in aerated BHI medium at 37°C. (B) Mortality of zebrafish embryos infected with each strain (n = 20–25 per group).
(TIF)

Figure S2 **The pattern of clonality observed during a systemic murine infection.** Pie charts show the distribution of three strains in each organ, for each mouse at each time point. Numbers inside rings indicate log_10_(CFU) in each organ (i.e. the total bacterial load). Where total load was <100 CFU, strain distribution is not given, as presenting data this close to the detection limit would be non-representative of true strain dominance. The mouse marked * was culled 6 hours earlier than the other mice in that group due to ill health and was not included in general analyses throughout the paper.
(TIF)

Figure S3 **The effect of tetracycline on zebrafish embryos infected with** ***S. aureus*** **SH1000 strains.** (A) Mortality of zebrafish infected with SH1000 EryR alone and treated with a range of tetracycline doses (n = 25–30 per group). (B) Terminal EryR bacterial load per EryR-infected embryo, treated with 2.5 µg/ml tetracycline. (C) Terminal EryR/TetR strain ratio per embryo infected with a 1∶1 mixture of SH1000 EryR∶TetR bacteria and treated with 1 µg/ml tetracycline. Solid lines indicate median values.
(TIF)

Figure S4 **The effect of tetracycline on growth of** ***S. aureus*** **SH1000** ***in vivo*** **.** Graphs show growth kinetics of (A) EryR and (B) TetR. Zebrafish embryos were either left untreated (circles) or treated with 2.5 µg/ml tetracycline (squares). Bacterial CFU loads in living fish (n = 5 per group per time-point) at 1 and 7 hours post infection and every 12 hours thereafter, were determined (white). Bacterial loads in any dead fish at each time-point were also determined (black).
(TIF)

Figure S5 **The effect of tetracycline on zebrafish embryos infected with** ***S. aureus*** **NewHG strains.** (A) Mortality of zebrafish infected with NewHG EryR alone, treated with a range of tetracycline doses (n = 30–35 per group). (B) Mortality of zebrafish infected with a 1∶1 mixture of NewHG EryR∶TetR, treated with 2.5 µg/ml tetracycline (n = 70–75 per group). (C) Total terminal CFU load per embryo infected with a 1∶1 mixture of NewHG EryR∶TetR, treated with 2.5 µg/ml tetracycline. (D) Terminal EryR/TetR strain ratio per *pu*.1 morphant (phagocyte-depleted embryo), treated with 10 µg/ml tetracycline. Solid lines indicate mean (C) and median (D) values.
(TIF)

Figure S6 **The effect of tetracycline on zebrafish embryos infected with** ***P. aeruginosa*** **PAO1 strains.** (A) Mortality of zebrafish inoculated with either live bacteria, heat-killed bacteria or sterile PBS (n = 30–35 per group). (B) Mortality of zebrafish infected with PAO1 GmR alone, treated with a range of tetracycline doses (n = 55–60 per group). (C) Mortality of zebrafish infected with a 1∶1 mixture of PAO1 GmR∶TetR, treated with 50 µg/ml tetracycline (n = 65–70 per group). (D) Total terminal CFU load per embryo infected with a 1∶1 mixture of PAO1-L GmR∶TetR, treated with 50 µg/ml tetracycline. (E) Terminal GmR/TetR strain ratio per *pu*.1 morphant (phagocyte-depleted embryo), treated with 50 µg/ml tetracycline. Solid lines indicate mean (D) and median (E) values.
(TIF)

Figure S7 **The effect of oxacillin on zebrafish embryos infected with** ***S. aureus*** **strains.** (A) Mortality of zebrafish infected with BH1CC *ΔmecA*::*tetR* (OxS) alone, treated with a range of oxacillin doses (n = 40–60 per treated group, n = 80 untreated). (B) Mortality of zebrafish infected with a 1∶1 mixture of BH1CC OxS∶OxR, treated with 32 µg/ml oxacillin (n = 65–70 per group). (C) Total terminal CFU load per embryo infected with a 1∶1 mixture of BH1CC OxS∶OxR, treated with 32 µg/ml oxacillin. (D) Terminal BH1CC OxS/OxR strain ratio per *pu*.1 morphant (phagocyte-depleted embryo), treated with 32 µg/ml oxacillin. (E) Terminal BH1CC OxS∶OxR strain ratio in zebrafish treated with 16 µg/ml oxacillin. (F) Mortality of zebrafish infected with either SH1000 or KC043 (*katA ahpC*), treated with a range of oxacillin doses (n = 20–22 per group). Solid lines indicate mean (C) and median (D, E) values.
(TIF)

Figure S8 **The effect of 100 µg/ml (sub-curing) erythromycin on zebrafish embryos infected with** ***S. aureus*** **SH1000 strains.** (A) Mortality of zebrafish infected with either SH1000 EryR or TetR individually (n = 80–90 per group). (B) Mortality of zebrafish infected with a 1∶1 mixture of SH1000 EryR∶TetR (n = 80 per group). (C) Total terminal CFU load per embryo infected with a 1∶1 mixture of SH1000 EryR∶TetR. Solid lines indicate mean values.
(TIF)

Figure S9 **The effect of sub-curative antibiotic doses on the pattern of** ***S. aureus*** **NewHG infection in different murine organs.** EryR/TetR strain ratio is given for (A) kidneys, (B) livers and (C) spleens at two days post infection. Only organs that contained bacterial CFU above the limit of detection are shown. Solid lines indicate median values.
(TIF)

Table S1 **Bacterial strains and plasmids used in this study.**
(DOCX)
