# Supplementary figures and images for: Clonal Expansion during Staphylococcus aureus Infection Dynamics Reveals the Effect of Antibiotic Intervention
Source: PLoS Pathog. 2014 Feb 27;10(2):e1003959. doi: 10.1371/journal.ppat.1003959 (PMC3937288; doi:10.1371/journal.ppat.1003959)

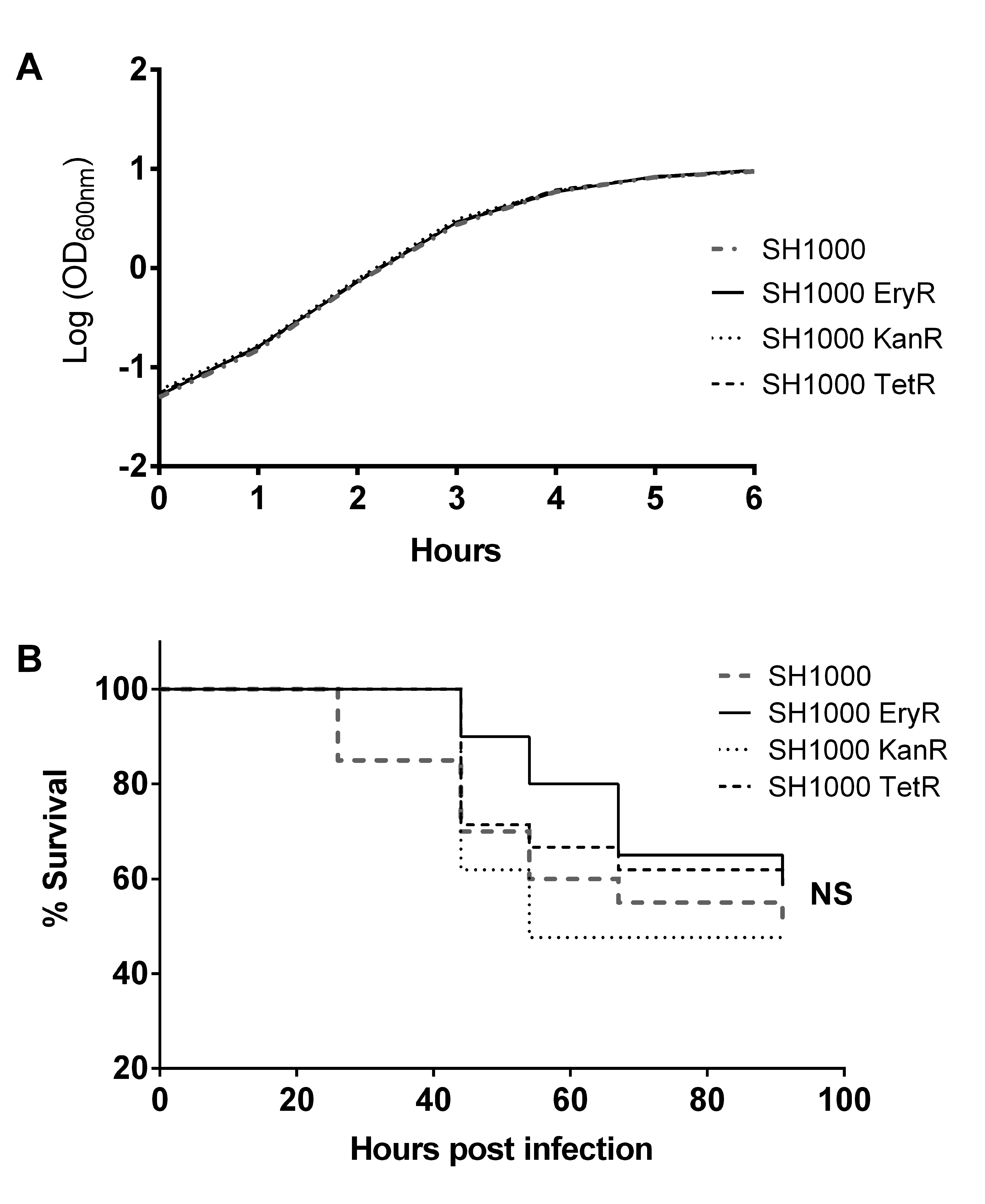

Supplement: Figure S1 — Comparison of fitness of antibiotic resistance-tagged SH1000 strains. (A) Growth in aerated BHI medium at 37°C. (B) Mortality of zebrafish embryos infected with each strain (n = 20–25 per group). (TIF) [file ppat.1003959.s001.tif]

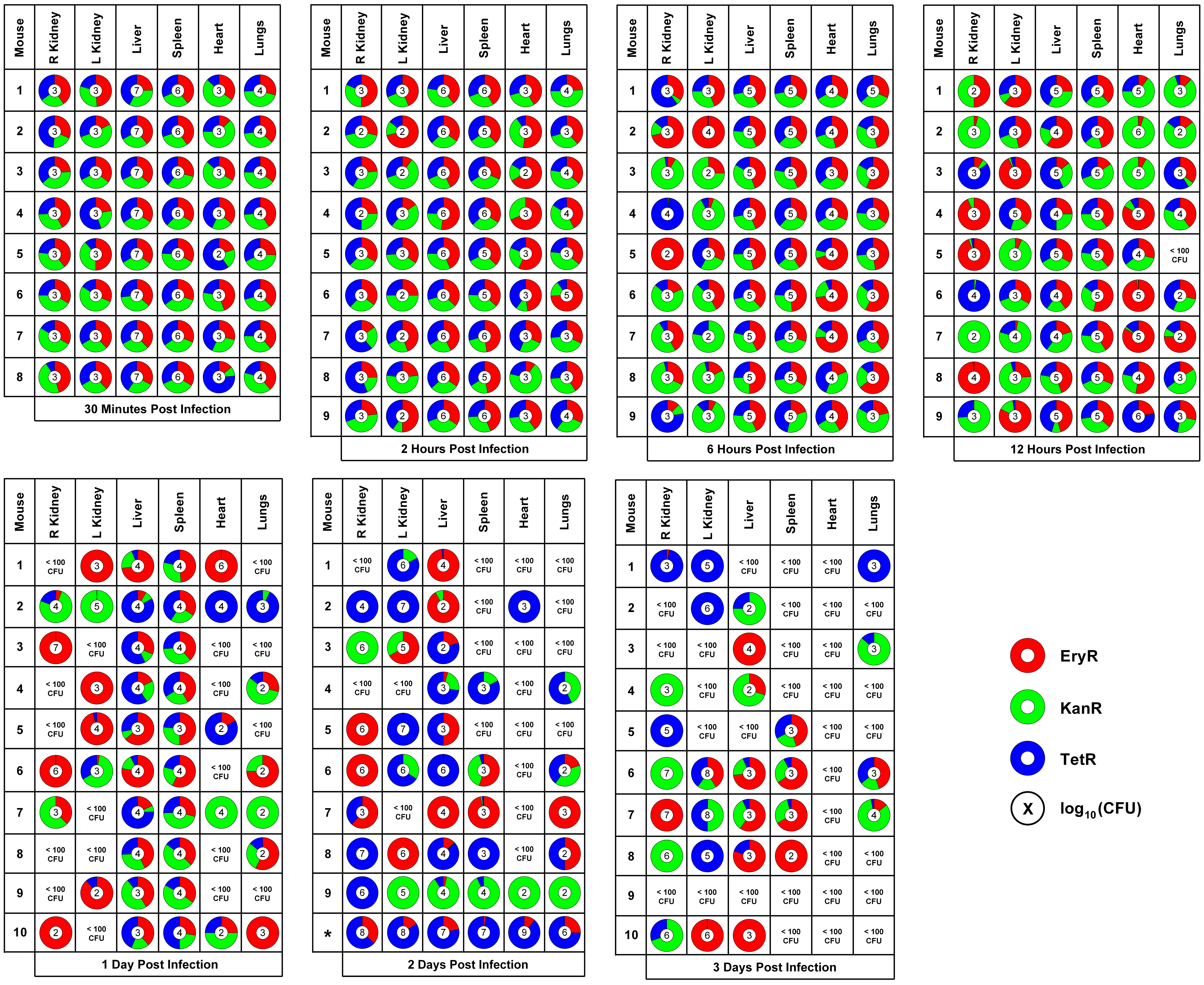

Supplement: Figure S2 — The pattern of clonality observed during a systemic murine infection. Pie charts show the distribution of three strains in each organ, for each mouse at each time point. Numbers inside rings indicate log10(CFU) in each organ (i.e. the total bacterial load). Where total load was <100 CFU, strain distribution is not given, as presenting data this close to the detection limit would be non-representative of true strain dominance. The mouse marked * was culled 6 hours earlier than the other mice in that group due to ill health and was not included in general analyses throughout the paper. (TIF) [file ppat.1003959.s002.tif]

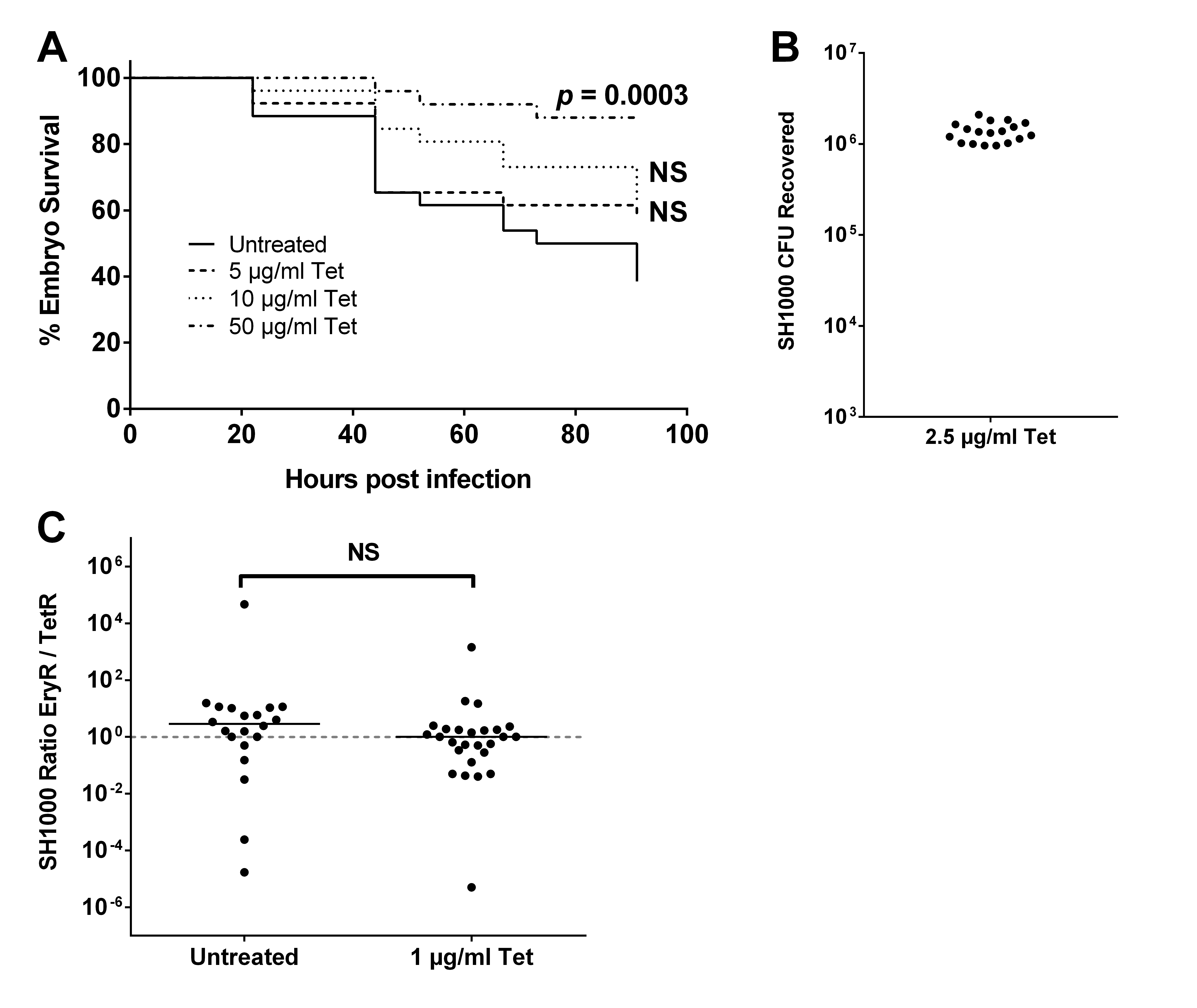

Supplement: Figure S3 — The effect of tetracycline on zebrafish embryos infected with S. aureus SH1000 strains. (A) Mortality of zebrafish infected with SH1000 EryR alone and treated with a range of tetracycline doses (n = 25–30 per group). (B) Terminal EryR bacterial load per EryR-infected embryo, treated with 2.5 µg/ml tetracycline. (C) Terminal EryR/TetR strain ratio per embryo infected with a 1∶1 mixture of SH1000 EryR∶TetR bacteria and treated with 1 µg/ml tetracycline. Solid lines indicate median values. (TIF) [file ppat.1003959.s003.tif]

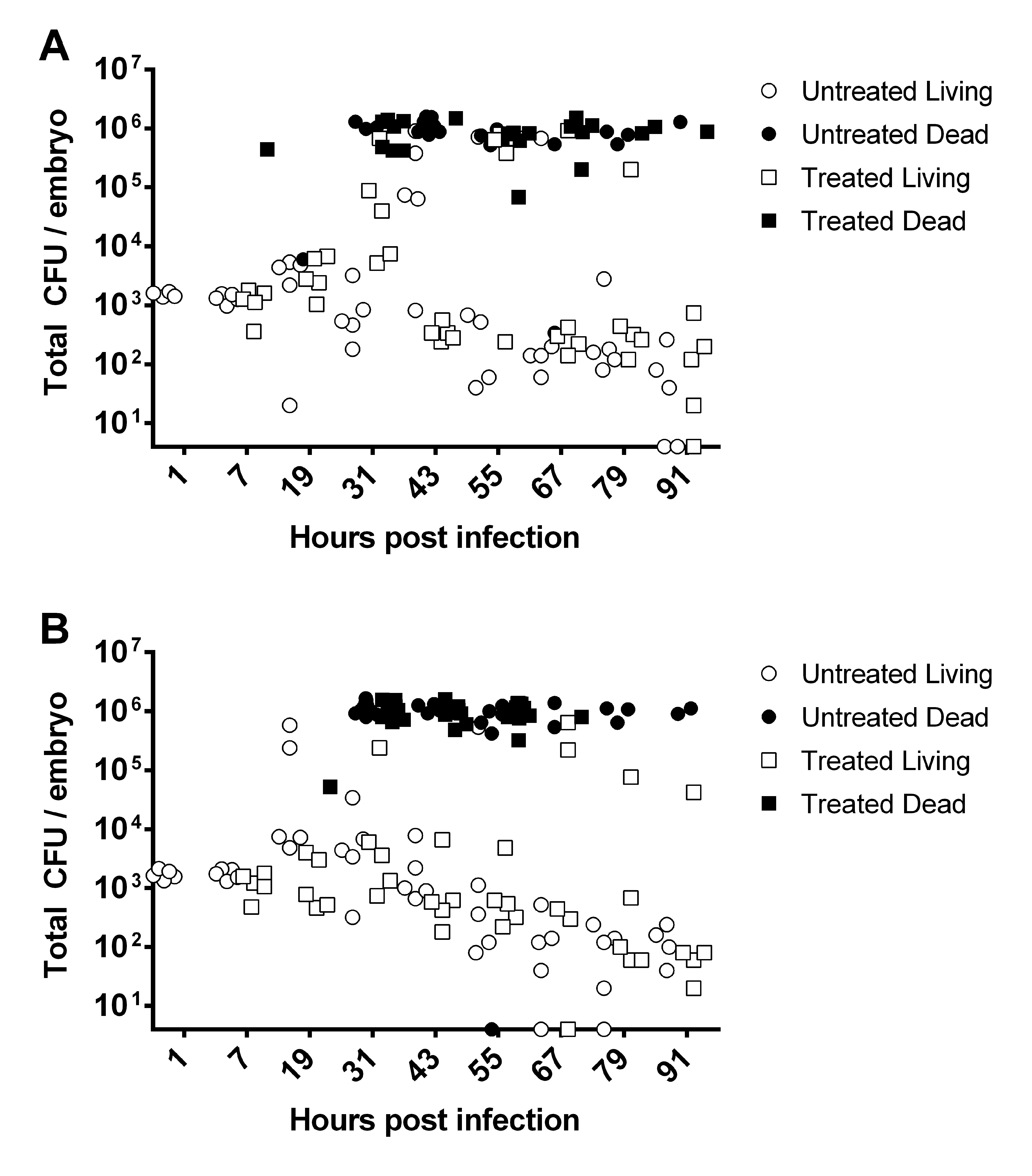

Supplement: Figure S4 — The effect of tetracycline on growth of S. aureus SH1000 in vivo . Graphs show growth kinetics of (A) EryR and (B) TetR. Zebrafish embryos were either left untreated (circles) or treated with 2.5 µg/ml tetracycline (squares). Bacterial CFU loads in living fish (n = 5 per group per time-point) at 1 and 7 hours post infection and every 12 hours thereafter, were determined (white). Bacterial loads in any dead fish at each time-point were also determined (black). (TIF) [file ppat.1003959.s004.tif]

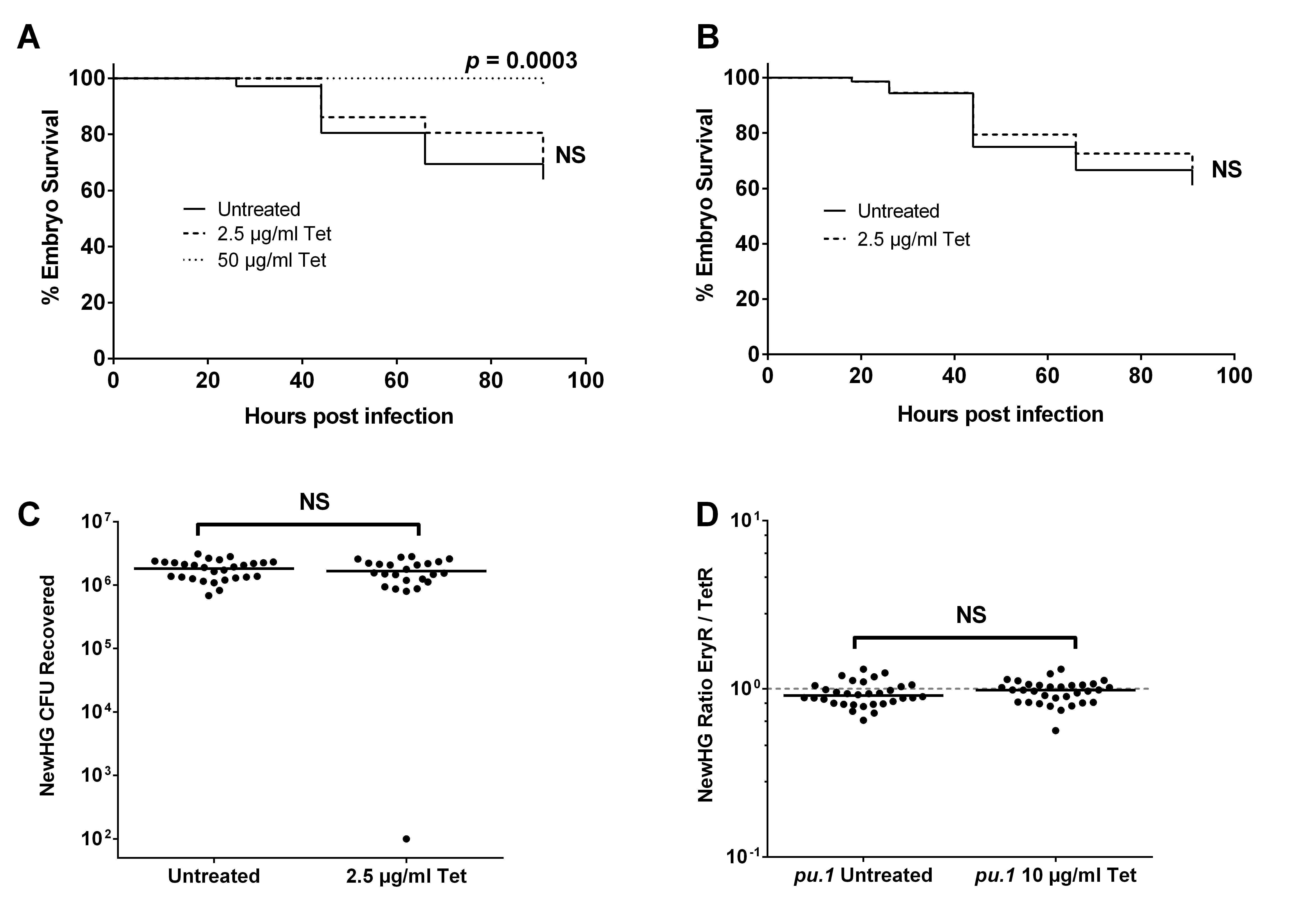

Supplement: Figure S5 — The effect of tetracycline on zebrafish embryos infected with S. aureus NewHG strains. (A) Mortality of zebrafish infected with NewHG EryR alone, treated with a range of tetracycline doses (n = 30–35 per group). (B) Mortality of zebrafish infected with a 1∶1 mixture of NewHG EryR∶TetR, treated with 2.5 µg/ml tetracycline (n = 70–75 per group). (C) Total terminal CFU load per embryo infected with a 1∶1 mixture of NewHG EryR∶TetR, treated with 2.5 µg/ml tetracycline. (D) Terminal EryR/TetR strain ratio per pu.1 morphant (phagocyte-depleted embryo), treated with 10 µg/ml tetracycline. Solid lines indicate mean (C) and median (D) values. (TIF) [file ppat.1003959.s005.tif]

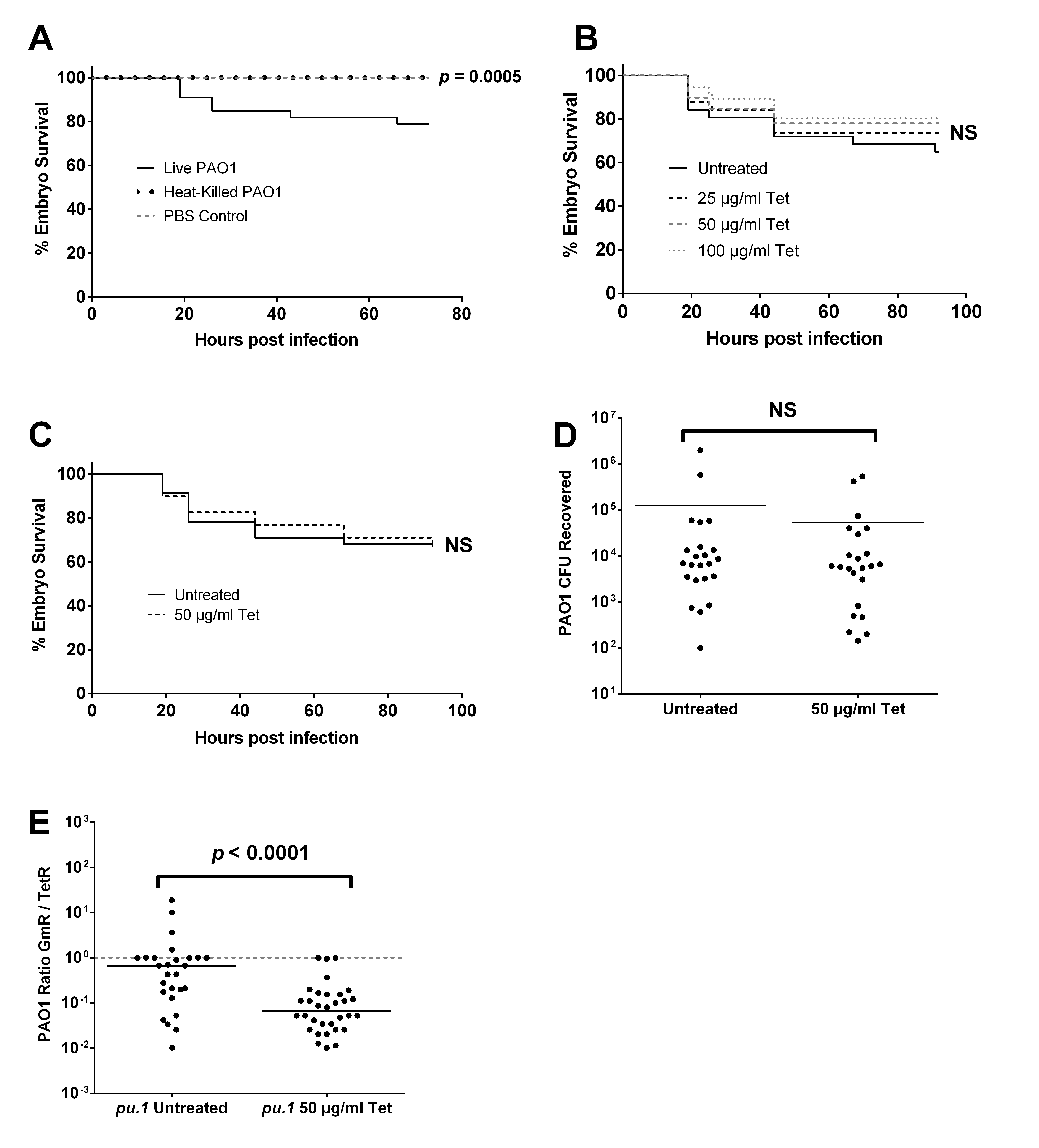

Supplement: Figure S6 — The effect of tetracycline on zebrafish embryos infected with P. aeruginosa PAO1 strains. (A) Mortality of zebrafish inoculated with either live bacteria, heat-killed bacteria or sterile PBS (n = 30–35 per group). (B) Mortality of zebrafish infected with PAO1 GmR alone, treated with a range of tetracycline doses (n = 55–60 per group). (C) Mortality of zebrafish infected with a 1∶1 mixture of PAO1 GmR∶TetR, treated with 50 µg/ml tetracycline (n = 65–70 per group). (D) Total terminal CFU load per embryo infected with a 1∶1 mixture of PAO1-L GmR∶TetR, treated with 50 µg/ml tetracycline. (E) Terminal GmR/TetR strain ratio per pu.1 morphant (phagocyte-depleted embryo), treated with 50 µg/ml tetracycline. Solid lines indicate mean (D) and median (E) values. (TIF) [file ppat.1003959.s006.tif]

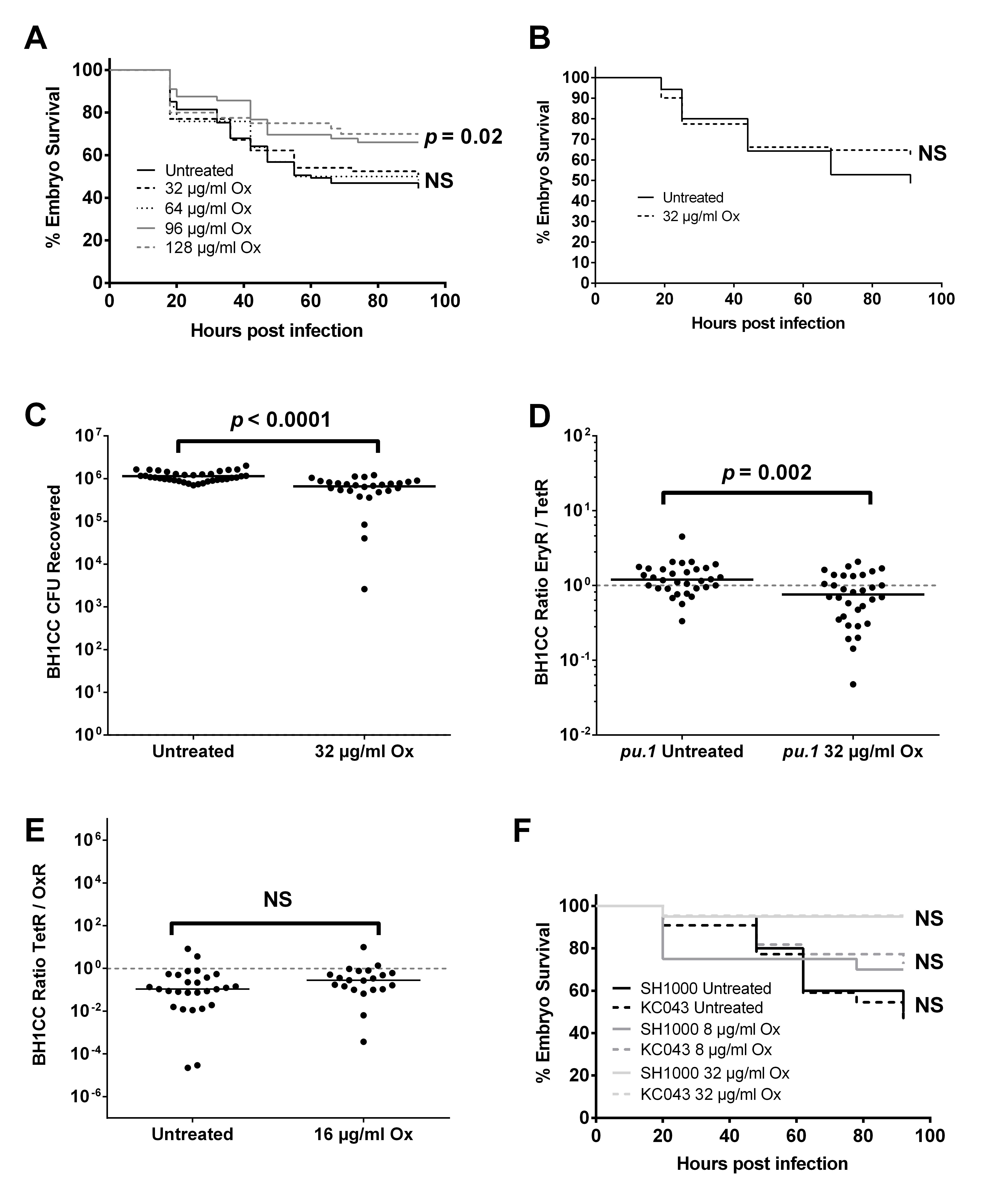

Supplement: Figure S7 — The effect of oxacillin on zebrafish embryos infected with S. aureus strains. (A) Mortality of zebrafish infected with BH1CC ΔmecA::tetR (OxS) alone, treated with a range of oxacillin doses (n = 40–60 per treated group, n = 80 untreated). (B) Mortality of zebrafish infected with a 1∶1 mixture of BH1CC OxS∶OxR, treated with 32 µg/ml oxacillin (n = 65–70 per group). (C) Total terminal CFU load per embryo infected with a 1∶1 mixture of BH1CC OxS∶OxR, treated with 32 µg/ml oxacillin. (D) Terminal BH1CC OxS/OxR strain ratio per pu.1 morphant (phagocyte-depleted embryo), treated with 32 µg/ml oxacillin. (E) Terminal BH1CC OxS∶OxR strain ratio in zebrafish treated with 16 µg/ml oxacillin. (F) Mortality of zebrafish infected with either SH1000 or KC043 (katA ahpC), treated with a range of oxacillin doses (n = 20–22 per group). Solid lines indicate mean (C) and median (D, E) values. (TIF) [file ppat.1003959.s007.tif]

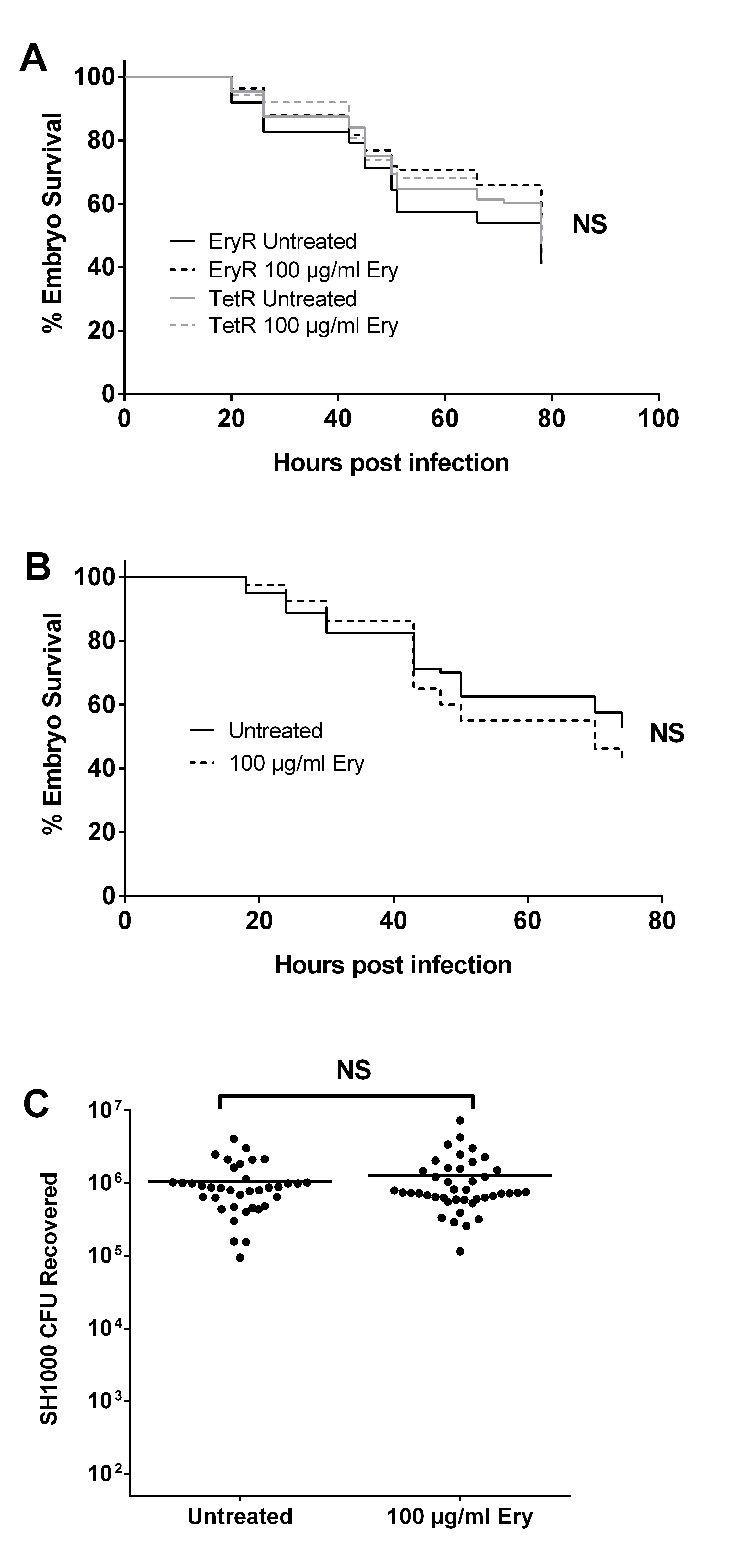

Supplement: Figure S8 — The effect of 100 µg/ml (sub-curing) erythromycin on zebrafish embryos infected with S. aureus SH1000 strains. (A) Mortality of zebrafish infected with either SH1000 EryR or TetR individually (n = 80–90 per group). (B) Mortality of zebrafish infected with a 1∶1 mixture of SH1000 EryR∶TetR (n = 80 per group). (C) Total terminal CFU load per embryo infected with a 1∶1 mixture of SH1000 EryR∶TetR. Solid lines indicate mean values. (TIF) [file ppat.1003959.s008.tif]

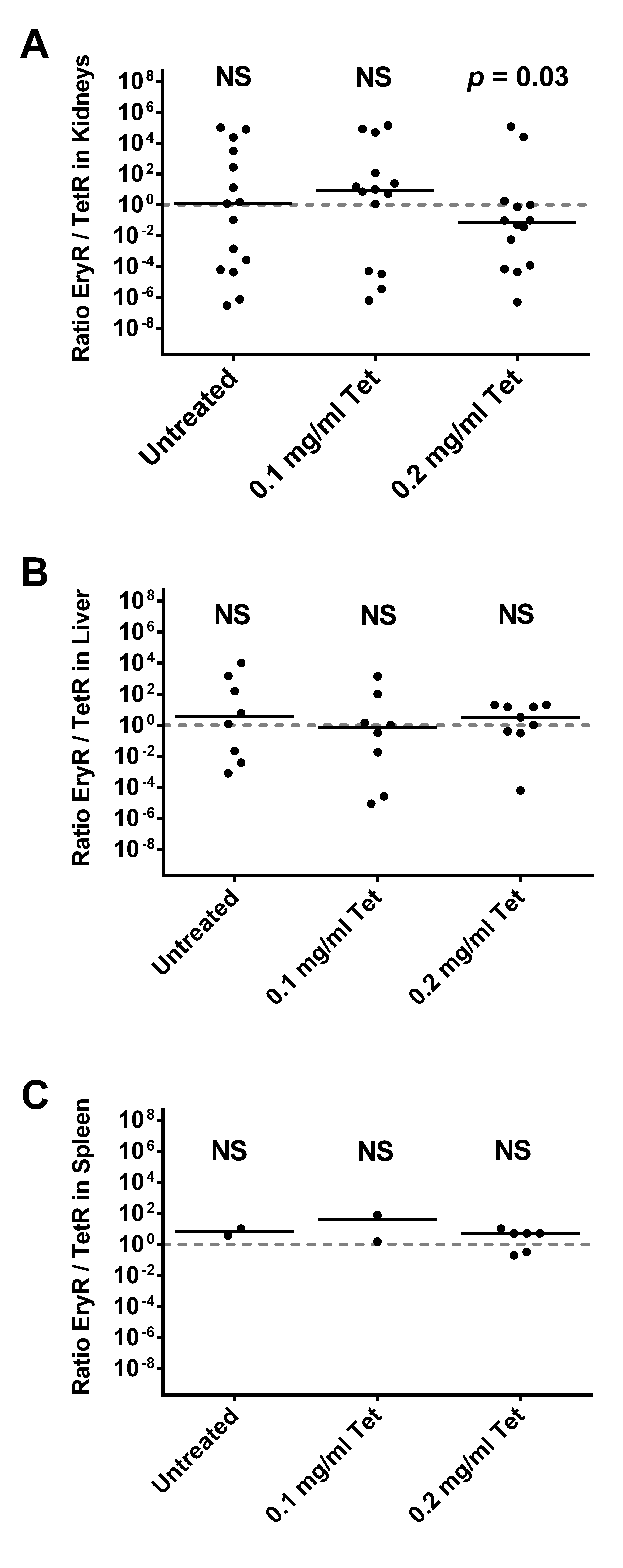

Supplement: Figure S9 — The effect of sub-curative antibiotic doses on the pattern of S. aureus NewHG infection in different murine organs. EryR/TetR strain ratio is given for (A) kidneys, (B) livers and (C) spleens at two days post infection. Only organs that contained bacterial CFU above the limit of detection are shown. Solid lines indicate median values. (TIF) [file ppat.1003959.s009.tif]
