# Supplementary material for: Clonal Expansion during Staphylococcus aureus Infection Dynamics Reveals the Effect of Antibiotic Intervention
Source: PLoS Pathog. 2014 Feb 27;10(2):e1003959. doi: 10.1371/journal.ppat.1003959 (PMC3937288; doi:10.1371/journal.ppat.1003959)
Supplement: Table S1 — Bacterial strains and plasmids used in this study. (DOCX) [file ppat.1003959.s010.docx]

**Table S1: Bacterial strains and plasmids used in this study.**

| Strain/Plasmid | Description | Reference |
| --- | --- | --- |
| SH1000 | Functional *rsbU*+ derivative of *S. aureus* 8325-4 | [63] |
| RN4220 | Restriction-deficient transformation recipient *S. aureus* | [64] |
| NewHG | *S. aureus* Newman with *saeS^L^* allele from strain RN1 | [32] |
| GMSA015 | SH1000 *lysA*::pGM068 (EryR) *lysA*+ | This study |
| GMSA016 | SH1000 *lysA*::pGM072 (KanR) *lysA*+ | This study |
| GMSA017 | SH1000 *lysA*::pGM070 (TetR) *lysA*+ | This study |
| GMSA021 | NewHG *lysA*::pGM068 (EryR) *lysA*+ | This study |
| GMSA022 | NewHG *lysA*::pGM072 (KanR) *lysA*+ | This study |
| GMSA023 | NewHG *lysA*::pGM070 (TetR) *lysA*+ | This study |
| KC043 | SH1000 *ahpC*::TetR *katA*::Tn*917* | [42] |
| PAO1-L | Wild type *Pseudomonas aeruginosa* | [65] |
| GMPA001 | PAO1-L mini-Tn7(GmR) | This study |
| GMPA002 | PAO1-L mini-Tn7(TetR) | This study |
| BH1CC | *S. aureus* MRSA isolate | [38] |
| BH1CCΔ*mecA* | BH1CC Δ*mecA*::TetR | [38] |
| pMUTIN4 | Cloning suicide vector (EryR) | [57] |
| pAISH1 | pMUTIN4 EryR- TetR+ | [58] |
| pGL433 | TAP-tag-KanR cassette | [59] |
| pGM068 | pMUTIN4 including *lysA* 3’ fragment | This study |
| pGM071 | pMUTIN4 EryR- KanR+ | This study |
| pGM070 | pAISH1 including *lysA* 3’ fragment | This study |
| pGM072 | pGM071 including *lysA* 3’ fragment | This study |
